# EHMTI-0180. Behavioural and alexithymic characteristics of adolescents suffering from chronic headache

**DOI:** 10.1186/1129-2377-15-S1-D39

**Published:** 2014-09-18

**Authors:** E Maffioletti, MM Mensi, E Antonaci, MA Chiappedi, F Ferro, S Molteni, F Galli, F Piazza, U Balottin

**Affiliations:** 1 Child Neuropsychiatry Unit, IRCCS National Neurological Institute "C. Mondino" - Pavia, Pavia, Italy; 2 Child Neuropsychiatry Unit, University of Pavia - Pavia, Pavia, Italy; 3 Child Neuropsychiatry Unit, IRCCS National Neurological Institute C. Mondino - Pavia, Pavia, Italy

## Introduction

many studies have examined the association between paediatric headache and psychopathology; some of them raised the possibility that headache frequency and severity could be worsened by a reduced psychological ability to mentally process emotions and affects.

## Aims

The first aim of this study was to assess psychopathological comorbidity in adolescents with chronic daily headache (CDH) compared to adolescents with non-chronic headache. The second aim was to investigate the possible role of alexithymia as a negative factor in adolescents with headache.

## Methods

42 patients aged 11.0-17.11, consecutively seen for headache in our Headache Centre, and their 42 mothers were enrolled. They were assessed using Parent Child Behaviour Checklist (CBCL), Youth Self-report (YSR) and Toronto Alexithymia Scale (TAS). A detailed history was taken to assess the presence of headache, using criteria defined by ICDH-III beta.

## Results

21 (50%) of our patients presented a form of CDH; they had at the YSR higher levels of Somatic Complaints (P=0.006), Thought Problems (P=0.003) and ADHD (P=0.049). At the CBCL, their mothers reported higher levels of Somatic Complaints (P=0.045) and lower Total Competences (P=0.012). Alexithymic patients showed more Social Problems (P=0.039), Thought Problems (P=0.010), Attention Deficit (P=0.024) and Affective Problems (P=0.036) compared to non-alexithymic patients.

## Conclusions

This study confirmed that CDH are associated with a higher level of impairment and with a heavier psychopathological burden. It is possible that the presence of a significant degree of alexythimia in these patients could be associated to the worsening both of headache and of psychopathological aspects.

No conflict of interest.

